# Genome-Wide Identification, Evolutionary Analysis and Seasonal Expression Patterns of the Sox Gene Family in the Daurian Ground Squirrel (*Spermophilus dauricus*)

**DOI:** 10.3390/vetsci13060553

**Published:** 2026-06-03

**Authors:** Zhuanxia Li, Zhiqing Feng, Hong Ye, Jia Li, Yufei Zan, Linjuan Wang, Fengcheng Song, Ziyang Liu, Wei Xia, Zhengrong Yuan, Xiuxiang Meng

**Affiliations:** 1 College of Biological Sciences and Technology, Beijing Forestry University, No. 35 Qinghua East Road, Haidian District, Beijing 100083, China; lzx18093243107@163.com (Z.L.); yufeizan@bjfu.edu.cn (Y.Z.); wanglinjuan@bjfu.edu.cn (L.W.); nkjmkksfc218@bjfu.edu.cn (F.S.); m19933648236@163.com (Z.L.); 2 Beijing Marco-Union Pharmaceutical, Beijing 100070, China; 13901090931@139.com; 3 School of Ecology and Environment, Renmin University of China, No. 59 Zhongguancun Street, Haidian District, Beijing 100872, China; 4 College of Animal Science and Technology, Hebei Agricultural University, Baoding 071000, China; lj18730066197@163.com (J.L.); xiaweihawaii@163.com (W.X.)

**Keywords:** Sox gene family, genome-wide analysis, Daurian ground squirrel, seasonal breeding, testis

## Abstract

The Sox (SRY-related high-mobility group box) gene family encodes transcription factors characterized by a conserved high-mobility group (HMG) box DNA-binding domain and plays essential roles in metazoan development, including sex determination, embryogenesis, neurogenesis, organogenesis, and reproductive regulation. Seasonal breeders provide valuable models for investigating reproductive regulation. The Daurian ground squirrel (*Spermophilus dauricus*) is a typical species for studying seasonal reproductive adaptation. In this study, Sox genes were identified at the genome-wide level in the Daurian ground squirrel. A total of 11 genes were identified and classified into 5 subfamilies, SoxC (*SdSox4*, *SdSox11*), SoxD (*SdSox5*, *SdSox6*, *SdSox13*), SoxE (*SdSox8*, *SdSox9*, *SdSox10*), SoxF (*SdSox17*, *SdSox18*), SoxG (*SdSox15*). Structural and evolutionary analyses revealed conserved HMG domains along with diversification among subfamilies. The genetic homology of the Sox family between the Daurian ground squirrel and the Thirteen-lined ground squirrel (*Ictidomys tridecemlineatus*) was discovered through collinearity analysis. Furthermore, hematoxylin and eosin (HE) staining revealed seasonal morphological changes in the testes of the adult Daurian ground squirrel. The seasonal expression patterns of 7 reproduction-related Sox genes were analyzed in testicular tissues. All analyzed genes exhibited significantly higher expression levels in the breeding season than the non-breeding season. Notably, *SdSox5*, *SdSox6*, *SdSox8*, and *SdSox13* showed pronounced seasonal differences in expression by the real-time quantitative polymerase chain reaction (RT-qPCR). This study provides new insights into the evolutionary characteristics and potential roles of Sox genes in seasonal reproductive regulation in mammals.

## Simple Summary

The Sox (SRY-related HMG-box) gene family plays essential roles in development, reproduction, and sex determination, but its functions in seasonal breeding mammals remain poorly understood. In this study, a total of 11 Sox genes were identified and characterized in the Daurian ground squirrel (*Spermophilus dauricus*), a typical seasonal breeder. Evolutionary analyses revealed conserved genomic features and high similarity to Sox genes in related ground squirrel species. Seasonal changes in testicular morphology and the expression patterns of reproduction-related Sox genes were also examined. Reproduction-related Sox genes were significantly upregulated during the breeding season, suggesting important roles in testicular function and seasonal reproductive regulation. These findings provide new insights into the evolution of the Sox gene family and its potential roles in seasonal reproductive regulation in mammals.

## 1. Introduction

The Sox (SRY-related high-mobility group box) gene family encodes a group of transcription factors characterized by a highly conserved high-mobility group (HMG) box domain. This domain mediates sequence-specific DNA binding, interaction with the DNA minor groove, and pronounced DNA bending [1,2,3]. By remodeling DNA architecture and coordinating transcriptional regulatory complexes, Sox proteins are critically involved in the regulation of gene expression [4,5]. Since their discovery through sequence homology with the mammalian sex-determining gene *Sry* (sex-determining-region on the Y chromosome), Sox family members have been classified into 11 subgroups and shown to participate in diverse developmental processes [6,7]. Notably, Sox genes are indispensable regulators of testicular development and function. *Sry* initiates the male sex-determination pathway and directs bipotential gonads toward testis development, while *Sox9* consolidates testicular differentiation and maintains Sertoli cell (SC) identity [8,9]. Beyond embryonic gonadogenesis, *Sox8* and *Sox9* are essential for sustaining postnatal spermatogenesis and male fertility. Collectively, these findings highlight the critical roles of the Sox gene family in regulating testicular growth, differentiation, and male reproductive function in mammals [10,11].

Seasonal breeding is a widespread adaptive reproductive strategy among wild mammals, enabling synchronization of reproductive activity with favorable environmental conditions [12]. The Daurian ground squirrel (*Spermophilus dauricus*) is a typical seasonal breeder with a restricted breeding period from April to May. This breeding period is followed by prolonged reproductive quiescence from June to the following March, during which hibernation occurs between October and March. Accordingly, the testes undergo pronounced seasonal morphological and functional changes between the breeding and non-breeding seasons [13]. During the breeding season, testicular recrudescence is evident, characterized by increased testis size, well-developed seminiferous epithelium, active spermatogenesis, and elevated steroid hormone secretion. In contrast, the non-breeding season is characterized by testicular regression and reproductive dormancy. Previous studies in ground squirrels have identified key molecules involved in seasonal reproduction, including androgen receptor (*Ar*), cytochrome P450 aromatase (*Cyp19a1*), and estrogen receptors (*Ers*) in reproductive tissues [14].

In seasonally breeding mammals, cyclic gonadal maturation, development, and regression are essential adaptive processes that ensure reproductive success under fluctuating environmental conditions. These dynamic changes are tightly regulated by the coordinated interaction of multiple pathways, including hormonal signaling, transcriptional regulation, and energy metabolism. Given their essential roles in gonadal development and spermatogenesis, Sox genes may exhibit dynamic expression patterns during seasonal reproductive transitions [15,16]. Therefore, investigating the regulatory roles of Sox genes in seasonal reproduction is expected to provide valuable insights into both the functional diversity of the Sox gene family and the molecular mechanisms underlying seasonal reproductive regulation.

In this study, a comprehensive genome-wide analysis of the Sox gene family was carried out in the Daurian ground squirrel. The molecular characteristics of Sox genes, including physicochemical properties and predicted subcellular localization, were systematically analyzed. In addition, phylogenetic relationships, gene structures, conserved domains and motifs, and chromosomal distributions were analyzed to clarify the evolutionary features of the Sox gene family. Hematoxylin and eosin (HE) staining of testicular tissues collected during different reproductive seasons revealed marked seasonal variations in testicular morphology. Furthermore, the seasonal expression patterns of Sox genes in testicular tissues were analyzed at the mRNA level using real-time quantitative polymerase chain reaction (RT-qPCR) to investigate their potential roles in testicular growth and development. These findings provide insights into the biological functions of Sox genes and contribute to understanding the molecular mechanisms underlying seasonal reproduction in the Daurian ground squirrel.

## 2. Materials and Methods

### 2.1. Genome-Wide Identification of Sox Genes

All genomic data of the Daurian ground squirrel used for Sox gene identification were retrieved from the National Genomics Data Center (NGDC) (https://ngdc.cncb.ac.cn/ accessed on 15 March 2026) under accession number PRJCA044807. The Hidden Markov Model (HMM) profile of the conserved HMG-box domain (PF00505) was downloaded from the Pfam database and used as a query to identify putative Sox genes using HMMER (v3.3.2) with an E-value cutoff of 1 × 10⁻⁵. Candidate protein sequences were further examined for the presence of the HMG-box domain using the Conserved Domain Database (CDD, v3.21) (https://www.ncbi.nlm.nih.gov/cdd/ accessed on 28 January 2026), Pfam [17], and SMART (http://smart.embl-heidelberg.de/index2.cgi accessed on 21 December 2025) database to confirm the presence of the complete HMG-box domain [18,19,20]. Non-redundant Sox protein sequences were obtained by sequence alignment and manual inspection using DNAMAN (v9.0) [21]. The annotations were further validated through BLAST + (version 2.15.0) searches in the NCBI non-redundant (NR) database. Physicochemical properties of the protein encoded by each validated gene, including molecular weight (MW), theoretical isoelectric point (pI), instability index (II), aliphatic index (AI), and grand average of hydropathicity (GRAVY), were determined using the ExPASy ProtParam tool (https://web.expasy.org/protparam/ accessed on 7 December 2025) [22]. Subcellular localization was predicted using the WoLF PSORT server (v0.2) (https://wolfpsort.hgc.jp/ accessed on 5 February 2026) [23].

### 2.2. Analysis of Structural Features

Conserved domains of Sox proteins were identified using the CDD (v3.21) and subsequently visualized with TBtools (v2.119) [24]. Conserved motifs of the 11 Sox proteins in the Daurian ground squirrel were predicted using MEME (v5.0), with motif widths ranging from 6 to 50 amino acids and a maximum of 10 motifs specified [25]. Motif distribution patterns were visualized using TBtools (v2.119). Gene structure information, including exon–intron organization and untranslated regions (UTRs), was extracted from genome annotation files in GFF format. The exon, intron, and UTR structures of Sox genes were then graphically displayed using TBtools (v2.119) according to their genomic lengths and relative positions.

### 2.3. Chromosomal Distribution and Structural Characterization of Sox Proteins

The chromosomal positions of all Sox genes in the Daurian ground squirrel were extracted from the reference genome annotation files. Gene distribution across chromosomes was visualized using TBtools (v2.119), and the number of Sox genes on each chromosome was quantified to assess gene abundance and identify potential gene clusters. Gene densities and distribution patterns among chromosomes were further compared to investigate possible local duplications or expansions. Secondary structures of Sox proteins from the Daurian ground squirrel were predicted using multiple algorithms to enhance reliability. PSIPRED (v3.3) (http://bioinf.cs.ucl.ac.uk/psipred/ accessed on 1 March 2026) [26] was employed to generate secondary structure predictions based on PSI-BLAST-derived position-specific scoring matrices. Additional predictions were obtained using JPred4 (https://www.compbio.dundee.ac.uk/jpred/ accessed on 5 January 2026), which integrates the JNet (v2.2) algorithm and UniRef90 profiles to assign consensus secondary structures [27]. Homologous protein structures were identified using the PDB database. (http://www.rcsb.org/ accessed on 11 February 2026) Protein tertiary structures were predicted using AlphaFold3 with default parameters, and the resulting models were visualized using PyMOL (v3.1). [28,29]. The presence and cleavage sites of signal peptides were predicted using SignalP (v6.0), which utilizes deep neural networks to distinguish signal peptides from transmembrane regions and accurately determine cleavage sites, providing both type and location information for secreted proteins [30].

### 2.4. Phylogenetic, Multiple Sequence Alignment, and Collinearity Analysis of the Sox Gene Family

The protein sequences of the Sox gene family from representative vertebrate species were retrieved from the NCBI database (https://www.ncbi.nlm.nih.gov/ accessed on 21 December 2025) for subsequent comparative and phylogenetic analyses. The phylogenetic tree was constructed from the Sox gene amino acid sequences of *Spermophilus dauricus*, *Ailuropoda melanoleuca*, *Bos taurus*, *Capra hircus*, *Cricetulus griseus*, *Danio rerio*, *Erinaceus europaeus*, *Homo sapiens*, *Ictidomys tridecemlineatus*, *Marmota*, *Meriones unguiculatus*, *Microtus ochrogaster*, *Mus musculus*, *Ovis aries*, *Sciurus carolinensis*, *Urocitellus parryii*, and *Ursus maritimus* (Table S1). Protein sequences were aligned using ClustalW (v2.1) [31] with default settings, followed by phylogenetic tree construction in MEGA (v11.0) [32], utilizing the Neighbor-Joining (NJ) method and Jones–Taylor–Thornton (JTT) model, supported by 1000 bootstrap replicates. Adjustments to the tree were made using the ITOL (v6) website (https://itol.embl.de/login.cgi accessed on 25 November 2025) [33]. Multiple sequence alignments of all predicted Daurian ground squirrel Sox protein HMG-box domain sequences were performed using the online ClustalW tool (v2.1) (http://www.ebi.ac.uk/Tools/msa/clustalw2/ accessed on 26 February 2026) with the default parameters, and then the alignments were also manually adjusted. Collinearity analysis of homologous Sox genes in the Daurian ground squirrel genome was performed using the MCScanX toolkit [34], and the resulting syntenic relationships were visualized using TBtools (v2.119).

### 2.5. Animal Tissue Collection

The adult male Daurian ground squirrel were captured in Hebei Province, China. All animal experimental procedures were performed in accordance with the Animal Care and Use Policy and were approved by the Ethics Committee of Beijing Forestry University (Approval No. EAWC_BJFU_2022005) and performed in accordance with institutional guidelines and the ARRIVE guidelines for animal research. Animals were euthanized by intraperitoneal injection of sodium pentobarbital (150 mg/kg), which induces rapid and painless loss of consciousness [35]. The testes were carefully excised from adult male Daurian ground squirrels. A portion of the testicular tissues was immediately frozen and stored at −80 °C for subsequent molecular analyses, while the remaining tissues were rinsed with ice-cold phosphate-buffered saline (PBS) and immediately fixed in 4% paraformaldehyde at 4 °C for histological examination.

### 2.6. Histological Analysis of the Testis

For each reproductive season, testes from three adult male individuals were collected and used as independent biological replicates for histological analysis. Fixed tissues were subsequently dehydrated through a graded ethanol series, cleared in xylene, and embedded in paraffin. Paraffin blocks were sectioned at 5 μm thickness using a rotary microtome. Sections were mounted on glass slides, deparaffinized, rehydrated through a descending ethanol series, and stained with Hematoxylin and eosin (HE) following standard protocols. Stained sections were observed under a light microscope to assess testicular morphology, including seminiferous tubule structure and spermatogenic cell organization [36].

### 2.7. RNA Sample Extraction and Real-Time Quantitative Polymerase Chain Reaction (RT-qPCR)

Total RNA was extracted from testicular tissues using TRIzol reagent (Invitrogen, Waltham, MA, USA) according to the manufacturer’s instructions. RNA integrity was verified by agarose gel electrophoresis, and concentration was measured using a spectrophotometer [37]. The expression levels of Sox genes were measured by the RT-qPCR in testicular tissues collected from breeding and non-breeding seasons. For each reproductive season, testicular tissues from three adult male Daurian ground squirrels were collected and used as independent biological replicates. In addition, three technical replicates were performed for each biological sample. For the RT-qPCR, specific primers were designed using Premier (v6.0) software and synthesized by Sangon Biotech (Shanghai, China). RNA concentration and integrity were assessed using a NanoDrop spectrophotometer (Thermo Fisher Scientific, located in Waltham, MA, USA) and agarose gel electrophoresis. The qualified RNA was reverse transcribed into cDNA using the All-in-One First-Strand Synthesis MasterMix kit (Lanbioed Technology, Shenzhen, China), and stored at −20 °C. The RT-qPCR was performed in 20 μL reactions using the FastStart DNA MasterPlus SYBR Green Kit (Roche, Mannheim, DE, USA) on an ABI PRISM 7500 Fast Real-Time PCR system (Applied Biosystems, Waltham, Massachusetts, USA) [38]. Each reaction contained SYBR Green master mix, gene-specific primers (Table 1), and cDNA template. Thermal cycling conditions were: 95 °C for 10 min; 40 cycles of 95 °C for 30 s, 60 °C for 30 s, and 72 °C for 30 s; with a final hold at 4 °C for storage. Melting curve analysis from 60 °C to 95 °C was conducted to confirm amplification specificity. *β-actin* was used as the internal reference gene. Relative expression levels were calculated using the 2^−ΔΔCT^ method and normalized to *β-actin* expression.

### 2.8. Statistical Analysis

Statistical analyses were performed using IBM SPSS Statistics (v31.0). Data are presented as mean ± SEM (standard error of the mean). Differences between breeding and non-breeding groups were analyzed using an independent-samples Student’s *t*-test. For RT-qPCR analysis, three biological replicates (independent animals) were included for each reproductive stage, and each sample was analyzed with three technical replicates. Technical replicates were averaged prior to statistical analysis. Differences were considered statistically significant at *p* < 0.05.

## 3. Results

### 3.1. Identification of Sox Genes in the Daurian Ground Squirrel Genome

A total of 11 Sox genes were identified in the Daurian ground squirrel genome. Based on phylogenetic classification, these genes were grouped into 5 subfamilies, including 2 SoxC (*SdSox4* and *SdSox11*), 3 SoxD (*SdSox5*, *SdSox6*, and *SdSox13*), 3 SoxE (*SdSox8*, *SdSox9*, *and SdSox10*), 2 SoxF (*SdSox18* and *SdSox17*), and 1 SoxG (*SdSox15*) (Table S1). The physicochemical properties of the Sox gene family in the Daurian ground squirrel were analyzed (Table 2). The protein lengths ranged from 233 amino acids (*SdSox15*) to 750 amino acids (*SdSox5*). Molecular weights ranged from 25572.87 (*SdSox15*) to 82426.83 (*SdSox5*) Da. The pI values of the proteins spanned 4.97 (*SdSox11*) to 9.77 (*SdSox15*). Among them, *SdSox11*, *SdSox17*, *SdSox8*, *SdSox9*, *SdSox10*, *SdSox5*, and *SdSox13* were categorized as acidic proteins (pI < 7.0), while *SdSox18*, *SdSox4*, *SdSox6*, *SdSox15* were classified as basic proteins (pI > 7.0). The instability index of all proteins exceeded 40, ranging from 52.44 (*SdSox4*) to 77.73 (*SdSox9*), indicating that these proteins may be predicted to be unstable under in vitro conditions. The aliphatic index varied from 47.96 (*SdSox9*) to 70.42 (*SdSox13*). Notably, *SdSox5*, *SdSox6*, and *SdSox13* showed relatively high thermostability, with aliphatic index values above 65. All proteins exhibited negative GRAVY values, ranging from −1.017 (*SdSox9*) to −0.468 (*SdSox4*), indicating that Sox proteins are predominantly hydrophilic in nature. Subcellular localization prediction indicated that all proteins are nuclear, consistent with their roles as transcription factors (Table 2).

### 3.2. Gene Structure and Conserved Motifs Analysis of Sox Genes

**Figure 1:**
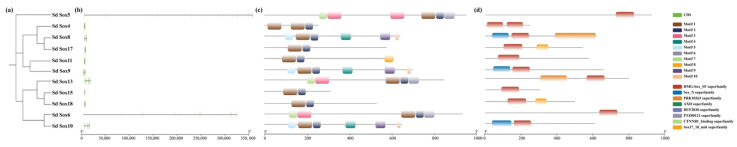
The schematic diagram of gene structure and conserved motif of Sox genes. (**a**) The phylogenetic tree was constructed using the Neighbor-Joining (NJ) method to illustrate the evolutionary relationships among the identified Sox gene family. (**b**) The gene structure diagram depicts the coding sequences (CDSs) as green rectangles and the introns as black lines. (**c**) Different functional domains are visualized as rectangles in distinct colors, with each color corresponding to a specific domain type. (**d**) Segments in various colors denote the predicted sequence motifs, and the legend on the right provides the corresponding motif identities.

Structural analyses of Sox genes included phylogenetic relationships (Figure 1a), coding sequence (CDS) organization, conserved motif distribution, and domain architecture. Gene structure analysis revealed considerable variation in exon–intron organization and in the number of CDS segments, which ranged from 1 to 14 among different Sox genes. Notably, *SdSox5* possessed the largest number of CDSs, whereas 2 SoxC members (*SdSox4* and *SdSox11*) had the fewest CDSs, indicating substantial structural divergence within the Sox gene family (Figure 1b). Conserved motif analysis identified 10 distinct motifs among all Sox proteins. Motifs 1 and 2 were present in all Sox proteins and corresponded to the conserved HMG-box domain. Although motif number and distribution varied among subfamilies, they were highly conserved within each group. For instance, the 3 SoxD members (*SdSox5*, *SdSox6*, and *SdSox13*) shared identical motif compositions, including motifs 1, 2, 3, 6, and 7 positioned at similar locations within the protein sequences. Similarly, 3 SoxE members (*SdSox8*, *SdSox9*, and *SdSox10*) contained 6 conserved motifs and uniquely possessed motifs 4, 5, 6, and 10, which were absent in other Sox proteins (Figure 1c). Conserved domain analysis further demonstrated that all Sox proteins contained the characteristic HMG domain, while the SoxN domain was detected exclusively in 3 SoxE members (*SdSox8*, *SdSox9*, and *SdSox10*), suggesting subfamily-specific functional specialization (Figure 1d).

### 3.3. Chromosomal Localization, Secondary Structure, Tertiary Structure Prediction, and Signal Peptide Analysis of Sox Gene Family

**Figure 2:**
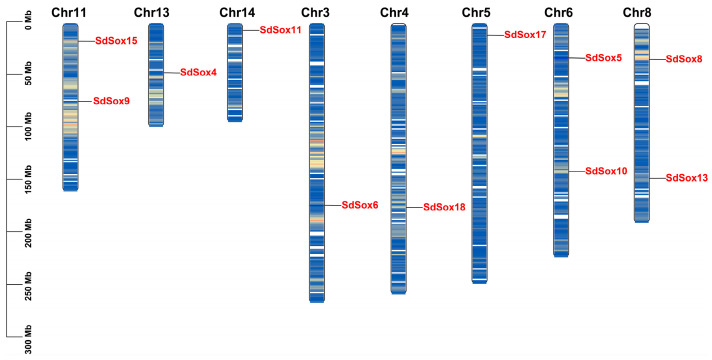
The Chromosomal distribution and density profile of the Sox gene family in Daurian ground squirrel. Each vertical bar represents an individual chromosome, with chromosome numbers indicated at the top. The scale on the left denotes the physical position along each chromosome, measured in megabase pairs (Mb). Red gene labels accompanied by horizontal tick marks indicate the precise genomic locations of individual *SdSox* gene family members on their respective chromosomes. The multicolored banding pattern along the chromosomes represents variation in genomic sequence features, specifically the distribution density of repetitive sequences across different chromosomal regions.

Chromosomal localization analysis revealed that the identified Sox genes in the Daurian ground squirrel genome were unevenly distributed across multiple chromosomes (Figure 2). Specifically, a total of 11 Sox genes were mapped to 8 different chromosomes—2 Sox genes were located on Chr6, and Chr8, and Chr11 whereas 1 gene was located each on Chr3, Chr4, Chr5, Chr13, and Chr14. Furthermore, these Sox genes were distributed at distinct loci along the chromosomes. For instance, *SdSox15* and *SdSox9* on Chr11 were separated by a relatively large genomic distance, and *SdSox5* and *SdSox10* on Chr6 were also located in distinct chromosomal regions (Figure 2).

**Figure 3:**
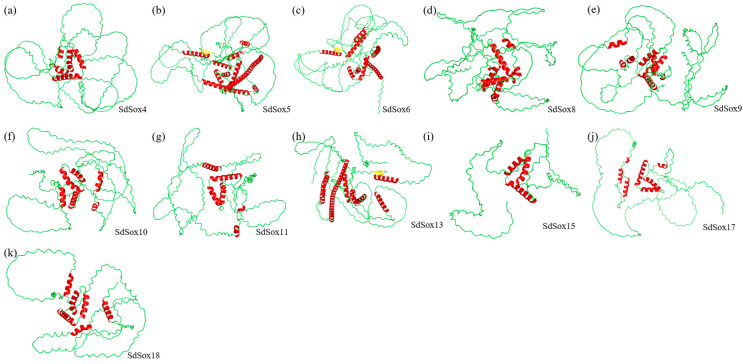
The predicted tertiary structures of Sox gene family in the Daurian ground squirrel. Three-dimensional (3D) structural models of Sox gene family were obtained with AlphaFold3 online software, and their corresponding images were displayed by PyMOL (3.1) software. The α-helixes, β-sheets, and coils in the images were colored by the rainbow adopted secondary structure succession pattern from the N-terminal to the C-terminal. The secondary structure elements include alpha helix, beta turn, and random coil (Panels (**a**–**k**) represent the proteins *SdSox4*, *SdSox 5*, *SdSox 6*, *SdSox 8*, *SdSox 9*, *SdSox 10*, *SdSox 11*, *SdSox 13 SdSox 15*, *SdSox 17*, *SdSox 18*).

Secondary structure prediction showed that Sox proteins mainly consisted of α-helices, random coils, β-turns, and extended strands. Overall, random coils constituted the predominant structural component in the most Sox proteins, with proportions ranging from 65.47% (*SdSox13*) to 86.73% (*SdSox9*). The α-helix was the second most abundant structural element, accounting for 11.49% (*SdSox9*) to 30.33% (*SdSox6*) of the total secondary structure. In contrast, β-turns and extended strands represented the least abundant components across all family members (Table S2, Figure 3). Signal peptide analysis was performed to evaluate the secretory characteristics of Sox family proteins in the Daurian ground squirrel. All analyzed proteins were classified as belonging to the “OTHER” category, corresponding to the absence of signal peptides. The predicted probabilities for the “OTHER” class were consistently close to 1 (0.999944–1), whereas those for other signal peptide types (Sec/SPI and Sec/SPII) were negligible (≤0.000092) or zero. These findings indicate that none of the Sox proteins in the Daurian ground squirrel contain signal peptides, suggesting that they function as non-secretory proteins (Table S3).

### 3.4. Phylogenetic Analysis, Multiple Sequence Alignment, and Collinearity Analysis

**Figure 4:**
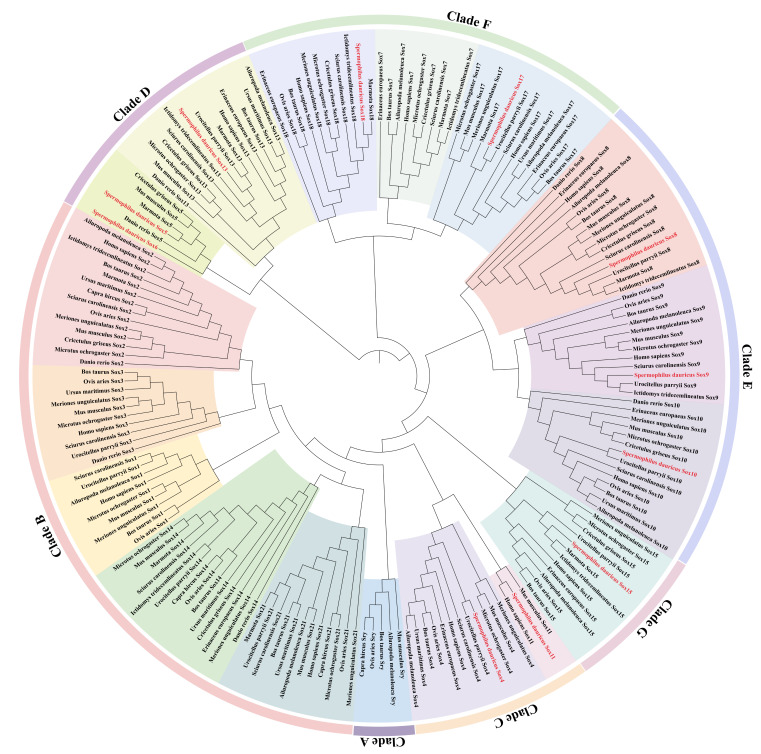
The phylogenetic tree of Sox proteins from 17 species. Phylogenetic tree of 185 Sox proteins from 17 species, including *Spermophilus dauricus*, *Ailuropoda melanoleuca*, *Bos taurus*, *Capra hircus*, *Cricetulus griseus*, *Danio rerio*, *Erinaceus europaeus*, *Homo sapiens*, *Ictidomys tridecemlineatus*, *Marmota*, *Meriones unguiculatus*, *Microtus ochrogaster*, *Mus musculus*, *Ovis aries*, *Sciurus carolinensis*, *Urocitellus parryii*, and *Ursus maritimus*. The distance tree was rooted by the lush orthologs. Branch support was estimated using 1000 bootstrap replicates, and bootstrap values were displayed with color circles at the branch nodes in different sizes.

A total of 185 Sox protein sequences that derived from 17 species were employed for phylogenetic tree construction, with each sequence assigned a corresponding accession number (Table S4). Phylogenetic analysis demonstrated that all Sox protein sequences were clearly resolved into 7 well-defined clades, corresponding to their canonical subfamilies, including Clades A, B, C, D, E, F, and G. Members within each subfamily formed distinct monophyletic groups, indicating a high degree of evolutionary conservation across vertebrate lineages (Figure 4)**.** Clade G contained a single member, *SdSox15*, which exhibited the closest phylogenetic relationship with *Sox15* from *Marmota* and *Ictidomys tridecemlineatus*. Clade C comprised 2 members, *SdSox4* and *SdSox11*, and this gene number was consistent with that observed in other mammalian species. Clade D included 3 members, *SdSox5*, *SdSox6*, and *SdSox13*. Among them, *SdSox6* and *SdSox13* clustered closely with their respective homologs from other mammals. In addition, *SdSox5* and *SdSox6* displayed similar evolutionary positions, suggesting shared evolutionary features within this subfamily. Clade F consisted of 2 members, *SdSox17* and *SdSox18*. However, *SdSox7*, which is commonly present in this clade in other vertebrates, was not identified in the Daurian ground squirrel genome in this study. In contrast, Clade E contained 3 members, and all typical representatives of this subfamily (*SdSox8*, *SdSox9*, and *SdSox10*) were successfully identified (Figure 4). Notably, no corresponding subfamily members of the Daurian ground squirrel were observed in Clades A and B, indicating that genes belonging to these 2 subfamilies were not identified in the genome data of the Daurian ground squirrel in this study. In contrast, members of all other subfamilies were successfully detected, suggesting the evolutionary conservation of these subfamilies in the Daurian ground squirrel.

**Figure 5:**
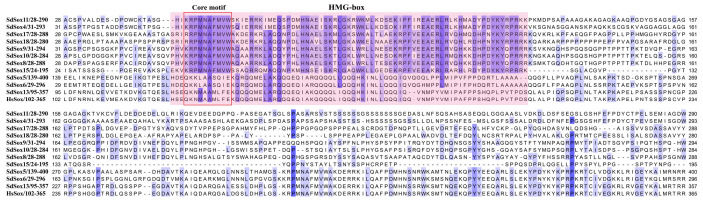
The sequence analysis of the high-mobility group (HMG) box domains of Sox proteins in the Daurian ground squirrel. The conserved HMG-box core motif is highlighted in red, with the canonical HMG-box region shaded in pink. Amino acid residues are numbered on the right. Acid residues that are identical among all Sox proteins and the amino acid residues with conservation rates greater than 75% are indicated in blue.

The multiple sequence alignment of HMG-box domains from the Daurian ground squirrel Sox proteins reveals a high level of sequence conservation (Figure 5). Amino acid residues that were identical across all sequences are highlighted in dark blue, whereas residues with a conservation rate greater than 75% are shown in lighter shades of blue and purple. This high conservation underscores the critical role of the HMG-box in recognizing and binding to the minor groove of DNA, a hallmark function of Sox transcription factors. Residues essential for the characteristic L-shaped three-dimensional structure of the HMG-box, including those involved in hydrophobic packing and DNA-interaction, are strictly conserved across all Daurian ground squirrel Sox proteins. This conservation ensures the structural stability and DNA-binding specificity of the domain, which is fundamental to the regulatory activity of Sox factors in processes such as embryonic development and cell fate determination (Figure 5).

**Figure 6:**
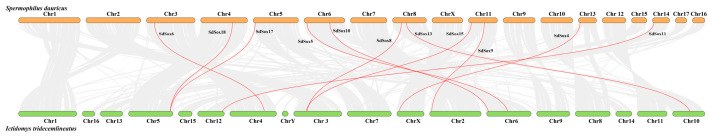
The collinearity analysis of Sox genes between Daurian ground squirrel and Thirteen-lined ground squirrel. Co-linear gene pairs are depicted with gray lines, whereas Sox gene pairs are marked in red.

Whole-genome collinearity analysis was performed to investigate the evolutionary conservation and genomic organization of the Sox gene family between the Daurian ground squirrel and the Thirteen-lined ground squirrel (Figure 6). In the collinearity map, homologous gene pairs between the two genomes are connected by gray lines representing large-scale syntenic blocks, whereas syntenic relationships involving Sox genes are highlighted in red to emphasize their conserved genomic associations. The analysis revealed that *SdSox17*, located on Chr5 of the Daurian ground squirrel, exhibited conserved synteny with its ortholog on the same chromosome in the Thirteen-lined ground squirrel. Similarly, *SdSox5* and *SdSox10*, positioned on Chr6 of the Daurian ground squirrel showed clear conserved synteny with their respective orthologs on the corresponding chromosome of the Thirteen-lined ground squirrel (Figure 6).

### 3.5. Histology Analysis

**Figure 7:**
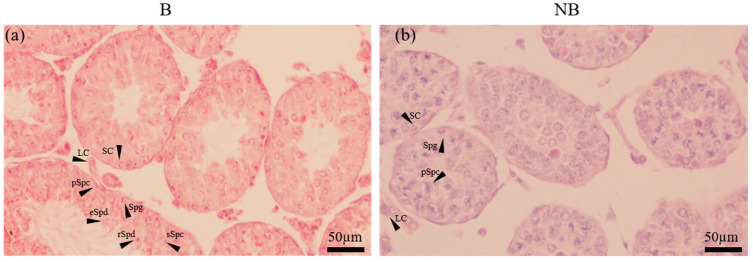
The Hematoxylin and eosin staining of seminiferous tubules of the wild male ground squirrels during the breeding season (B) and non-breeding season (NB). Panels (**a**,**b**) represent staining in the breeding and non-breeding seasons, respectively. Abbreviations: SC, Sertoli cell; LC, Leydig cell; Spg, spermatogonia; pSpc, primary spermatocyte; sSpc, secondary spermatocyte; rSpd, round spermatid; eSpd, elongated spermatid. The scale bar represents 50 μm.

To investigate the morphological characteristics of the testes in wild male Daurian ground squirrel during the breeding season and the non-breeding season, testicular tissues were subjected to HE staining. The results showed that, during the breeding season, the seminiferous tubules exhibited intact and well-organized structures, with enlarged and regular luminal diameters and a thick, densely packed seminiferous epithelium (Figure 7). Spermatogenic cells were arranged in well-defined layers, and a complete spermatogenic cell lineage was clearly observed, including spermatogonia (Spg) located adjacent to the basement membrane, primary spermatocytes (pSpc), secondary spermatocytes (sSpc), as well as round spermatids (rSpd) and elongated spermatids (eSpd) near the lumen, indicating active and complete spermatogenesis in the testes. SC displayed intact morphology and were evenly distributed within the seminiferous epithelium, and morphologically normal Leydig cells (LC) were observed in the interstitial tissue (Figure 7a). In contrast, during the non-breeding season, the seminiferous tubules exhibited marked degenerative changes, characterized by significantly reduced luminal diameters and a markedly thinned seminiferous epithelium with loosely arranged and disorganized cells (Figure 7b). Spermatogenesis appeared largely inhibited, with sparse spermatogonia and primary spermatocytes present, and no obvious secondary spermatocytes, round spermatids, or elongated spermatids observed. No mature sperm cells were observed in the lumen, and the seminiferous epithelium displayed pronounced regressive morphological features. These results revealed marked seasonal variations in testicular morphology and spermatogenic activity in the Daurian ground squirrel. Active spermatogenesis and well-organized seminiferous epithelium were observed during the breeding season, whereas epithelial atrophy and spermatogenic arrest occurred during the non-breeding season.

### 3.6. Expression Patterns of Sox Genes

**Figure 8:**
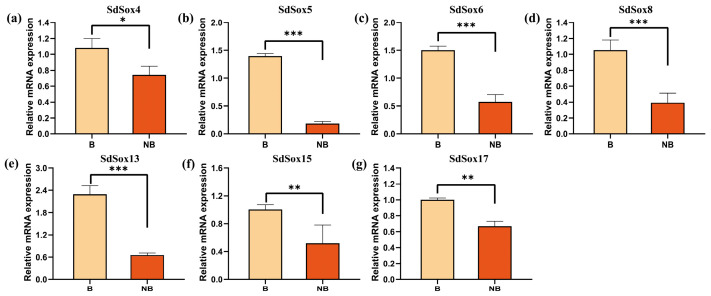
The mRNA expression of Sox genes detected by real-time quantitative polymerase chain reaction (RT-qPCR) in testes of the Daurian ground squirrel during breeding and non-breeding seasons (**a**) Relative mRNA expression of *SdSox4*; (**b**) Relative mRNA expression of *SdSox5*; (**c**) Relative mRNA expression of *SdSox6*; (**d**) Relative mRNA expression of *SdSox8*; (**e**) Relative mRNA expression of *SdSox13*; (**f**) Relative mRNA expression of *SdSox15*; (**g**) Relative mRNA expression of *SdSox17*.） (* *p* < 0.05, ** *p* < 0.01, and *** *p* < 0.001).

To investigate the molecular basis of seasonal testicular plasticity in the Daurian ground squirrel, mRNA expression levels of seven Sox genes (*SdSox4*, *SdSox5*, *SdSox6*, *SdSox8*, *SdSox13*, *SdSox15*, and *SdSox17*) were quantified in testicular tissues collected during the breeding season and non-breeding season using RT-qPCR. Across all analyzed genes, all genes showed significantly higher expression in the breeding season than in the non-breeding season (Figure 8a–g). Although all genes exhibited seasonal upregulation, the magnitude of expression changes varied among different Sox family members. Collectively, all 7 Sox family and related genes analyzed exhibited significantly higher mRNA expression in the testes of breeding-season compared to non-breeding-season in the Daurian ground squirrel, with changes ranging 1.5–8-fold, underscoring their coordinated role in seasonal testicular activation. Specifically, *SdSox5* and *SdSox13* showed the highest upregulation (7–8-fold and 4–5-fold, respectively), implicating them as key effectors of testicular functional remodeling and seminiferous epithelium maturation. *SdSox6* and *SdSox8* were also strongly upregulated (>3-fold and 2.8-fold, respectively), consistent with their conserved roles in regulating late-stage spermatogenesis and SC function. Moderate increases were observed for *SdSox4* (1.7-fold), *SdSox15* (2-fold), and *SdSox17* (1.5-fold).

## 4. Discussion

Sox genes encode a family of transcription factors characterized by a highly conserved HMG-box DNA-binding domain, which play crucial roles in cell fate determination, cellular differentiation, and developmental regulation across metazoans [39]. The Sox gene family is widely distributed across vertebrate genomes, with gene numbers varying among species []. For example, 27 Sox genes have been reported in both Nile tilapia and Zebrafish, 25 in Pufferfish, 19 in Medaka, 20 in Humans, 10 in the Florida lancelet, and 18 in the Western clawed frog [10,40]. In some teleost lineages, several Sox family members, such as *Sox12*, *Sox15*, *Sox16*, and *Sox30*, appear to be absent, suggesting lineage-specific gene loss or evolutionary divergence [41]. To date, over 20 Sox genes have been identified in vertebrates and classified into distinct subfamilies based on the conserved HMG-box and phylogenetic analyses []. *Sox1* is highly conserved with *Sox1A*, *Sox1B*, *Sox2*, and *Sox3*, all present in Humans [42], Brown rat [43], Red junglefowl [44]. Although over 20 Sox genes are typically identified in most vertebrates, only 11 Sox family members were detected in the current Daurian ground squirrel genome assembly, with several canonical Sox genes (*Sox1*, *Sox2*, *Sox3*, *Sox7*, *Sox14*, *Sox21*, and *Sox30*) not identified despite repeated multi-method searches and validation, and these genes were not excluded during the filtering process. Given the high conservation of the Sox gene family across vertebrates, the simultaneous absence of these key members is unlikely due to lineage-specific gene loss but more probably attributed to limitations of the current draft-level genome assembly and annotation, which can impair gene prediction accuracy especially for conserved transcription factor families like Sox genes.

Seasonal reproduction is an important life-history strategy that enables many species to adapt to environmental fluctuations [45]. Seasonal breeding is typically accompanied by cyclical testicular remodeling, which involves dynamic changes in testicular size, spermatogenic activity, germ cell composition, and endocrine function [46]. Seasonal reproductive patterns have been documented in numerous mammalian species, including the Daurian ground squirrel, Thirteen-lined ground squirrel, Giant panda, and Sheep [47,48,49]. These observations indicate that seasonal reproduction is a tightly regulated biological process controlled by complex molecular mechanisms rather than a passive physiological response to environmental variation. Due to its well-defined reproductive cycle, clear physiological transitions, and strong responsiveness to environmental cues, the Daurian ground squirrel represents an advantageous natural model for investigating the genetic and molecular mechanisms underlying mammalian seasonal reproduction [13,47,50]. Previous studies have shown that numerous genes and hormonal regulators involved in reproductive function display distinct seasonal expression patterns, including luteinizing hormone (*LH*), follicle-stimulating hormone (*FSH*), and genes associated with their signaling pathways [51,52].

In this study, a genome-wide identification and characterization of the Sox gene family was conducted in the Daurian ground squirrel, providing insights into the evolutionary and molecular features of this important transcription factor family. The identified Sox genes exhibited diverse physicochemical properties and predicted subcellular localization patterns, suggesting functional divergence among family members. Phylogenetic reconstruction, together with analyses of gene structures, conserved domains, motifs, and chromosomal distributions, further revealed that Sox genes in the Daurian ground squirrel share conserved evolutionary features with those reported in other mammals, while also displaying certain lineage-specific characteristics. Importantly, the seasonal expression profiles obtained from testicular tissues indicated that several Sox family members exhibit dynamic transcriptional changes across reproductive stages, indicating that these genes are likely involved in regulating testicular development and spermatogenesis. Given the unique seasonal reproductive strategy of the Daurian ground squirrel, such expression patterns suggest that Sox genes may contribute to the molecular regulatory network underlying seasonal testicular activity. Collectively, these findings improve our understanding of the evolutionary conservation and functional diversity of the Sox gene family and provide a theoretical foundation for future investigations into the molecular mechanisms underlying seasonal reproduction in this species.

### 4.1. Structural Features of Sox Family Proteins and Genes

The Sox gene family is highly evolutionarily conserved while also exhibiting functional diversification, as evidenced by variation in gene structure and protein organization. Despite this overall conservation, Sox genes display substantial variation in exon–intron organization and CDS number. Similar structural diversity has also been reported in mice and humans, suggesting that intron gain or loss events may contribute to lineage-specific regulatory complexity while preserving core protein functions [53,54]. Notably, *SdSox4* and *SdSox11* contain a single CDS, representing structurally compact genes that mayenable more efficient transcriptional regulation. Such compact gene architectures are frequently observed in transcription factors involved in tightly regulated developmental processes. While motif number and arrangement varied across Sox groups, motif composition was highly conserved within each subfamily, indicating subfamily-specific functional specialization. Conserved motif analysis was conducted, and it was revealed that motifs 1 and 2 were universally present in all Sox proteins. These motifs correspond to the HMG DNA-binding domain, which is essential for sequence-specific DNA recognition and transcriptional regulation [55]. For example, SoxD subfamily members (*SdSox5*, *SdSox6*, and *SdSox13*) shared identical motif arrangements, whereas SoxE subfamily members (*SdSox8*, *SdSox9*, and *SdSox10*) harbored unique motif combinations absent in other Sox proteins.

Protein structural analyses provided further insights into the potential functional properties of Sox family members. Secondary structure predictions revealed that random coils and alpha helices dominate Sox protein architecture, a feature commonly observed in transcription factors that require structural flexibility for DNA binding and interaction with cofactors [56]. The predominance of random coils may facilitate conformational adaptability, enabling Sox proteins to recognize diverse target sequences and participate in multiple regulatory complexes. Importantly, tertiary structure models generated by AlphaFold3 revealed that all Sox proteins share a conserved core architecture centered on the HMG-box DNA-binding domain, which forms a stable helix-turn-helix-like structure responsible for sequence-specific DNA recognition. Despite this conserved core, notable structural variations were observed among Sox subfamilies, particularly in the N-terminal and C-terminal regions as well as in surface-exposed loop regions. These differences may influence protein–protein interactions, chromatin accessibility, and transcriptional regulatory specificity, thereby contributing to functional diversification within the Sox gene family. Signal peptide prediction analysis demonstrated that all Sox family and related proteins lack classical signal peptides, confirming their non-secretory nature. This finding is consistent with their predicted nuclear localization and transcriptional regulatory roles [57]. Together with subcellular localization analyses, these results further support the conclusion that Sox proteins in the Daurian ground squirrel primarily function as intracellular regulators controlling gene expression programs rather than participating in extracellular signaling pathways.

Collectively, the conserved gene structures, motif architectures, and domain compositions of Sox genes underscore their evolutionary stability, while subfamily-specific structural features highlight functional diversification. These characteristics likely underpin the critical roles of Sox transcription factors in development, differentiation, and reproductive regulation. Given the pronounced seasonal reproductive physiology of the Daurian ground squirrel, the structural and functional diversity observed within the Sox gene family may contribute to fine-tuned transcriptional regulation underlying seasonal testicular remodeling and spermatogenesis, providing a molecular basis for adaptive reproductive strategies in this species.

### 4.2. Evolutionary Characterization of the Sox Gene Family

The Sox proteins identified in the Daurian ground squirrel exhibited considerable diversity in amino acid length, molecular weight, and isoelectric point, a pattern consistent with observations in other mammals such as Mouse, Humans, and Bovine [5,58]. All Sox proteins exhibited negative GRAVY values, indicating a predominantly hydrophilic nature. Such hydrophilicity is commonly observed in transcription factors and may facilitate interactions with DNA, cofactors, and chromatin-associated proteins in the nuclear environment. Furthermore, all Sox proteins were predicted to be unstable in vitro based on their instability index values (> 40). Similar instability has been reported for Sox proteins in mouse and human, where intrinsic disorder contributes to transcriptional plasticity and dynamic regulatory interactions [39]. However, the predicted instability in vitro does not necessarily indicate reduced functional stability under physiological conditions. As transcription factors, Sox proteins often undergo rapid turnover and transient regulation, which may facilitate precise temporal and spatial control of downstream target genes in response to developmental, physiological, and seasonal cues. The aliphatic index analysis further suggested differential thermostability among Sox family members. Proteins such as SdSox5, SdSox6, and SdSox13 exhibited relatively high aliphatic index values, comparable to their orthologs in Thirteen-lined ground squirrel and Marmot [59]. All Sox family proteins were predicted to localize to the nucleus and lacked signal peptides, confirming their non-secretory nature. This finding is fully consistent with previous reports across vertebrates, in which Sox proteins function as nuclear transcription factors binding to the minor groove of DNA via their HMG-box domains. The conserved nuclear localization across species highlights the evolutionary constraint acting on Sox genes to preserve their core regulatory functions.

Whole-genome synteny analysis between the Daurian ground squirrel and Thirteen-lined ground squirrel revealed extensive conservation of Sox gene loci, with most Sox genes exhibiting clear one-to-one orthologous relationships and collinear chromosomal positions. Such strong syntenic conservation suggests that Sox genes have been maintained under strong purifying selection during rodent evolution, consistent with previous comparative genomic studies in mammals [60]. Given the seasonal reproductive cycle of the Daurian ground squirrel, the preservation of these conserved genomic architectures may be crucial for precise temporal regulation of gene expression. Unlike tandemly duplicated gene families, Sox genes in the Daurian ground squirrel are distributed across eight chromosomes, a pattern also observed in mouse and human genomes. The absence of large Sox gene clusters suggests that segmental duplication has played a limited role in the expansion of the Sox family in this species. Furthermore, the overall dispersed distribution likely reflects long-term genome stability rather than recent duplication events [56].

Phylogenetic analysis based on Sox protein sequences from 17 representative species clearly resolved Sox genes into 7 canonical clades, consistent with established Sox classification systems. Most Sox subfamilies identified in other mammals were also present in the Daurian ground squirrel, highlighting strong evolutionary conservation. Interestingly, Sox genes belonging to Clades A and B were not detected in the Daurian ground squirrel genome. Similar gene losses have been reported in certain mammalian lineages and may reflect lineage-specific evolutionary events or functional redundancy compensated by other Sox members. In contrast, the complete retention of Clade E members (*SdSox8*, *SdSox9*, and *SdSox10*) is noteworthy, given their essential roles in neural crest development, glial differentiation, and gonadal development. This conservation suggests that these genes may play particularly important roles in the reproductive and developmental biology of ground squirrels. The absence of *SdSox7* from Clade F further suggests species-specific gene loss or divergence. *Sox7* is involved in vascular and endodermal development in other mammals, and its absence which may suggest potential functional compensation by related Sox members.

### 4.3. Regulatory Roles of Sox Genes in Seasonal Reproduction and Germ Cell Differentiation

The Sox gene family plays crucial roles in a wide variety of developmental contexts [61]. In the developing mouse gonads, *Sry* and *Sox9* play instructive roles in male sex determination and fetal testis development. In addition, *Sox4* contributes to fetal gonad development by regulating morphogenesis and germ cell differentiation. Furthermore, Sox4 is involved in the regulation of male fetal germ cell development [62]. In the present study, HE staining was performed to observe the cell types in the testes of ground squirrels during the breeding and non-breeding seasons. The results showed that all types of cells in the testes of ground squirrels were intact during the breeding season, with active spermatogenesis and a regular spermatogenic epithelium structure. Meanwhile, mRNA expression analysis revealed that the expression level of *Sox4* was significantly higher during the breeding season than in the non-breeding season. These findings indicate that *Sox4* plays a crucial role in regulating germ cell development in the Daurian ground squirrels, which is consistent with the conclusions of previous studies.

Seasonal breeders provide a powerful natural model for investigating reversible testicular plasticity and the molecular mechanisms that govern cyclic activation and regression of male reproductive function [55]. In the present study, quantitative expression profiling revealed that all examined Sox family and Sox-related genes exhibited significantly higher mRNA expression levels in the testes of breeding-season of Daurian ground squirrels compared with the non-breeding season. This coordinated increase in Sox gene expression during the breeding season suggests that Sox family members may act as important transcriptional regulators associated with the activation of spermatogenic and somatic cell functions during testicular recrudescence. Similar expression patterns of reproductive regulatory genes have been reported in other seasonally breeding mammals, including sheep and mice, where testicular recrudescence is accompanied by activation of germ cell proliferation, SC support, and androgen-dependent signaling pathways [63,64]. In contrast, the non-breeding period in Daurian ground squirrels is characterized by testicular regression, reduced spermatogenic activity, and suppressed endocrine function [13]. The markedly lower expression of Sox genes during this phase likely reflects transcriptional quiescence of spermatogenic and supporting somatic cell populations. Among the analyzed genes, *SdSox5* and *SdSox13* displayed the most pronounced seasonal upregulation, suggesting that they may serve as primary drivers of testicular structural and functional reactivation. In mice, *Sox5* is highly expressed in post-meiotic germ cells and has been implicated in chromatin remodeling and spermiogenesis. *Sox13*, although less studied in the testis, belongs to the SoxD group and has been linked to transcriptional regulation during cell differentiation [65].

## 5. Conclusions

This study provides a comprehensive genome-wide characterization of the Sox gene family in the Daurian ground squirrel, a representative seasonal breeding mammal. A total of 11 Sox genes were identified and classified into distinct subfamilies, revealing both evolutionary conservation and lineage-specific diversification. Phylogenetic, structural, and chromosomal analyses indicate that Sox genes have undergone differential evolutionary trajectories associated with functional specialization. The pronounced seasonal expression patterns of representative Sox genes in the testes suggest their involvement in testicular remodeling, spermatogenesis, and reproductive regulation. Overall, these findings expand our understanding of Sox gene evolution and function and provide a molecular basis for elucidating the genetic regulation of seasonal reproduction in mammals.

## Figures and Tables

**Table 1:** The real-time quantitative polymerase chain reaction (RT-qPCR) primers.

Gene Name	Primer
*SdSox4*	F: GCAAGATCATGGAGCAGTCG
	R: GAAGGTTAAGTCCGGCAACG
*SdSox5*	F: GACCCTTACCCTGTTCAGCT
	R: ACCTTGGTGCTGCTGTATCT
*SdSox6*	F: AGTTTACGGGAGCAGCTCTT
	R: GATCCAGGTTCAGGGTCACA
*SdSox8*	F: GTGGCACCATGTACAAGGC
	R: CGCCAGAACATCGACTTCAG
*SdSox13*	F: CAGCCCACTCAGAGCAAAAG
	R: ATGATCCCAGCTTTCCCTCC
*SdSox15*	F: GAGAGGTTGCCAAGAGTCCT
	R: CTCTGAGATTTCCAAGCGCC
*SdSox17*	F: TCATGGTGTGGGCTAAGGAC
	R:TCCCAACTACAAGTACCGGC
*β-actin*	F:CATTGAGCATGGCATCGTCA
	R:ATACATGGCTGGGGTGTTGA

**Table 2:** The physicochemical properties and subcellular localization of Sox gene family in the Daurian ground squirrel.

Gene Name	Number of Amino Acid	Molecular Weight	Theoretical pI	Instability Index	Aliphatic Index	Grand Average of Hydropathicity	Subcellular Localization
*SdSox4*	468	46,669.13	7.2	52.44	56.13	−0.468	Nucleus
*SdSox5*	750	82,426.83	6.38	62.74	68.75	−0.74	Nucleus
*SdSox6*	676	74,906.7	8.91	59.73	65.01	−0.789	Nucleus
*SdSox8*	463	49,360.07	6.68	59.74	50.71	−0.792	Nucleus
*SdSox9*	505	55,693.51	6.37	77.73	47.96	−1.017	Nucleus
*SdSox10*	468	50,037.25	6.12	59.19	53.01	−0.824	Nucleus
*SdSox11*	442	46,916.76	4.97	65.7	58.87	−0.686	Nucleus
*SdSox13*	614	68,240.06	6.02	77.35	70.42	−0.737	Nucleus
*SdSox15*	233	25,572.87	9.77	77.02	52.02	−0.8	Nucleus
*SdSox17*	418	44,501.99	5.91	64.27	58.49	−0.608	Nucleus
*SdSox18*	383	41,079.71	7.57	75.8	64.44	−0.577	Nucleus

## Data Availability

The data described in this article can be searched for and freely and openly accessed on the NCBI website (https://www.ncbi.nlm.nih.gov/ accessed on 25 November 2025).

## Supplementary Materials

The following supporting information can be downloaded at: https://www.mdpi.com/article/10.3390/vetsci13060553/s1, Table S1: The statistics of the species represented in the phylogenetic tree and the corresponding total number of Sox gene family; Table S2: The secondary structure prediction of Sox proteins; Table S3: The prediction of signal peptides and cleavage sites of Sox proteins; Table S4: The accession no. of different Sox gene family in different species.
